# Niche partitioning as a mechanism for locally high species diversity within a geographically limited genus of blastoid

**DOI:** 10.1371/journal.pone.0197512

**Published:** 2018-05-16

**Authors:** Ryan FitzGerald Morgan

**Affiliations:** Department of Chemistry, Geosciences, & Physics, Tarleton State University, Stephenville, Texas, United States of America; Duke University Marine Laboratory, UNITED STATES

## Abstract

*Deltoblastus batheri* and *Deltoblastus delta* occur concurrently in many Permian deposits from Timor. Closely related sister species living in direct proximity without alteration in feeding habit would be in direct violation of Lotka-Volterra dynamics. These two species were measured and compared to see if any evidence of differentiation along feeding lines has occurred in order to reduce direct competition. *P*-values obtained via Student’s t test display significant differentiation across all measured parameters. Thin-plate splines were used to visualize these differences, and clearly show the differences which are focused on the ambulacral region of the blastoids, which are the primary food gathering point for these species.

## Introduction

*Deltoblastus* Fay [1] is a speciose genus of spiraculate blastoid, known only from the Permian beds of Timor and nearby regions. The Timorese deposits are particularly diverse, with 18 described species occurring in concurrent beds [1–5]. The overlap in ranges and locations between the various *Deltoblastus* species makes this genus an ideal platform for studying complex biological processes, such as niche partitioning, among macroinvertebrates. Niche differentiation is the process by which competing populations use resources within the local environment, reducing direct competition between species allowing both to coexist [6]. While observed in the Recent, this process is challenging to observe and quantify in the fossil record. Particular challenges are the lack of well-constrained chronologies and continuous, or near-continuous, deposition and preservation of the fossils in question. *Deltoblastus*, with many species, pristine preservation, contemporaneous species, and limited geographic and chronostratigraphic extent, presents an ideal platform for observing niche differentiation in the fossil record. Niche partitioning is not unheard of in the fossil record, and has been suggested for other closely related species sharing ecospaces, including corals [7], crinoids [8; 9], sponges [10], and other fossil marine invertebrates [11–13]. Some authors have hypothesized that initial coexistence of closely related or sister species leads over a period 10 to 50 x 10^6^ years to macroevolution up to the family level and geographic differentiation [14]. Particular challenges are the lack of well-constrained chronologies and continuous, or near-continuous, deposition and preservation of the fossils in question. *Deltoblastus*, with many species, pristine preservation, contemporaneous species, and limited geographic and chronostratigraphic extent, presents an ideal platform for observing niche differentiation in the fossil record.

## Materials

**Fig 1:**
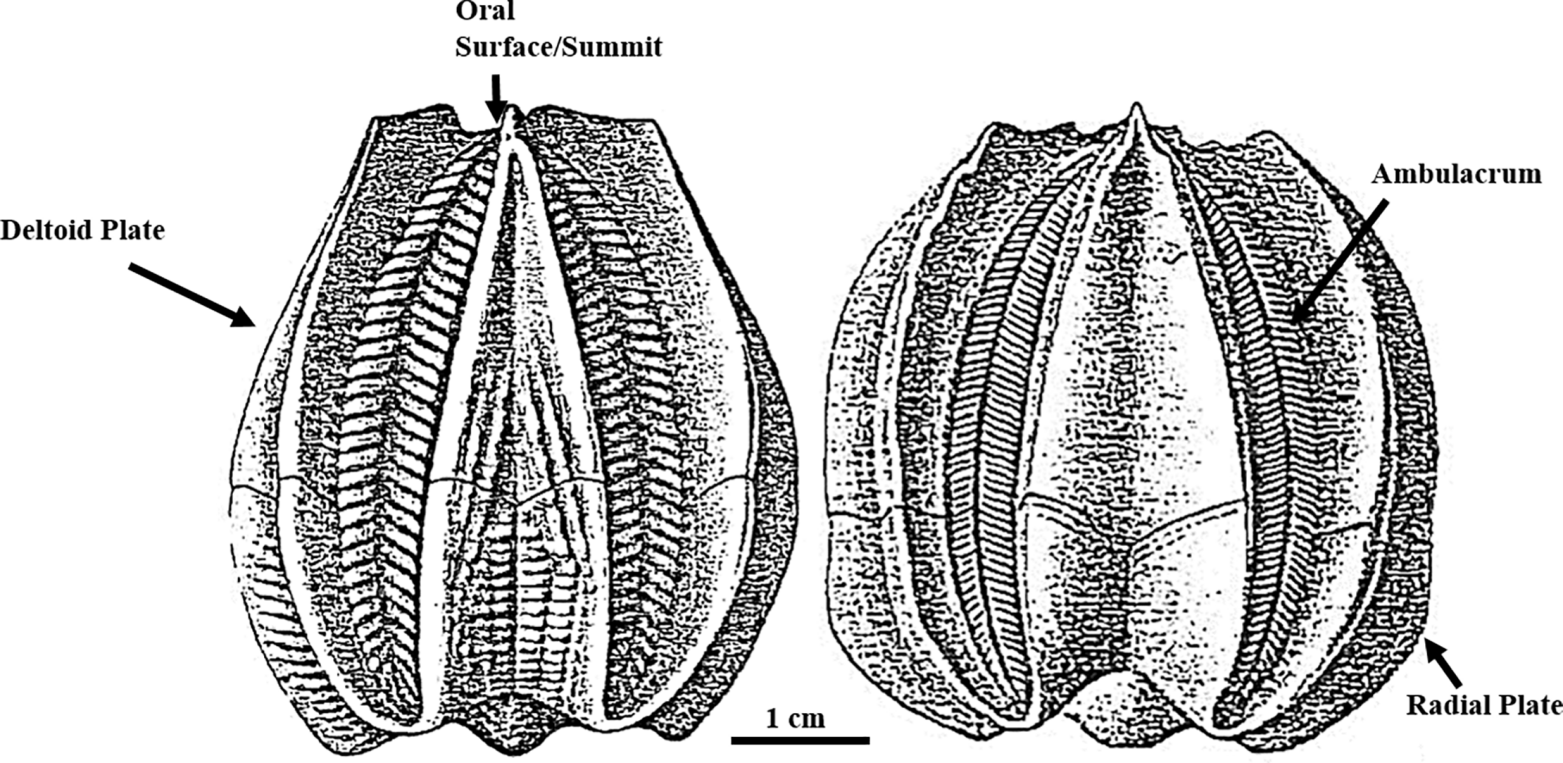
**Ambulacra view of *Deltoblastus batheri* (left) and *Deltoblastus delta* (right).** Adapted from past works [2].

While many of the *Deltoblastus* species could have been used, this study focused on *Deltoblastus batheri* and *Deltoblastus delta*, as these two species commonly occur together in large numbers (Fig 1), [5]. These two species also have been shown to possess extremely similar morphologies [5], with 19 of 30 measured characters matching in past analyses. In addition, these two species are readily available in many major museum collections, making the results of this analysis easily tested for validity. Measurements were taken from specimens housed within the Natural History Museum of London, United Kingdom (NMUK). These included 68 specimens of *D*. *batheri* and 365 specimens of *D*. *delta*, chosen for their completeness and lack of damage.

## Geologic setting

*Deltoblastus* is found in rocks exposed from along the southern and eastern Tethys, and range from early to middle Permian [15–17]. In Timor, the host sediments are typically carbonates and are hypothesized to represent open shelf to lagoon deposits [15]. The presence of nearby volcanics of similar age, along with the discontinuous sediment deposits, and seismic data demonstrating the region is underthrust by the Australian plate, suggests this region was an island arc which has since then undergone faulting upon collision with the Australian craton [15].

## Methodology

Measurements of *D*. *batheri* and *D*. *delta* were taken of select theca points using digital calipers, and compiled in a master sheet by measurement and specimen. Sixty-eight specimens in NMUK collections of *D*. *batheri* were deemed suitable for research, as 365 specimens of *D*. *delta*. Care was used to ensure accuracy of measurements, and all specimen identifications and associated information were noted. Averages were calculated of these measured data for the two species (Table 1). All specimens measured are publicly deposited and available for study from NMUK, located in London, United Kingdom. No permits were required for this study to be performed.

**Table 1:** Average thecal measurements (in mm) for *D*. *batheri* and *D*. *delta* with calculated variance and *t*-test results. Low *p*-values were calculated for all compared characters, indicating significant difference.

Morphofeature	*D*. *batheri* (n = 68)	*D*. *delta* (n = 365)	P value (Student’s t test)
**Average Height**	**17.981 +/- 3.1028**	**14.873 +/- 11.7955**	**p<<0.001**
**Average Width**	**14.926 +/- 2.95845**	**13.072 +/- 5.735**	**p<<0.001**
**Ambulacral Length**	**16.117 +/- 3.0234**	**13.141 +/- 11.201**	**p<<0.001**
**Ambulacral width**	**3.3049 +/- 0.16177**	**2.9528 +/- 0.3153**	**p<<0.001**
**Deltoid Length**	**12.561 +/- 1.54525**	**11.541 +/- 5.2655**	**p<<0.001**
**Radio-deltoid Width**	**4.8904 +/- 0.48547**	**4.3113 +/- 0.95845**	**p<<0.001**
**Radial Length**	**7.4606 +/- 0.79235**	**5.971 +/- 1.84625**	**p<<0.001**

**Fig 2:**
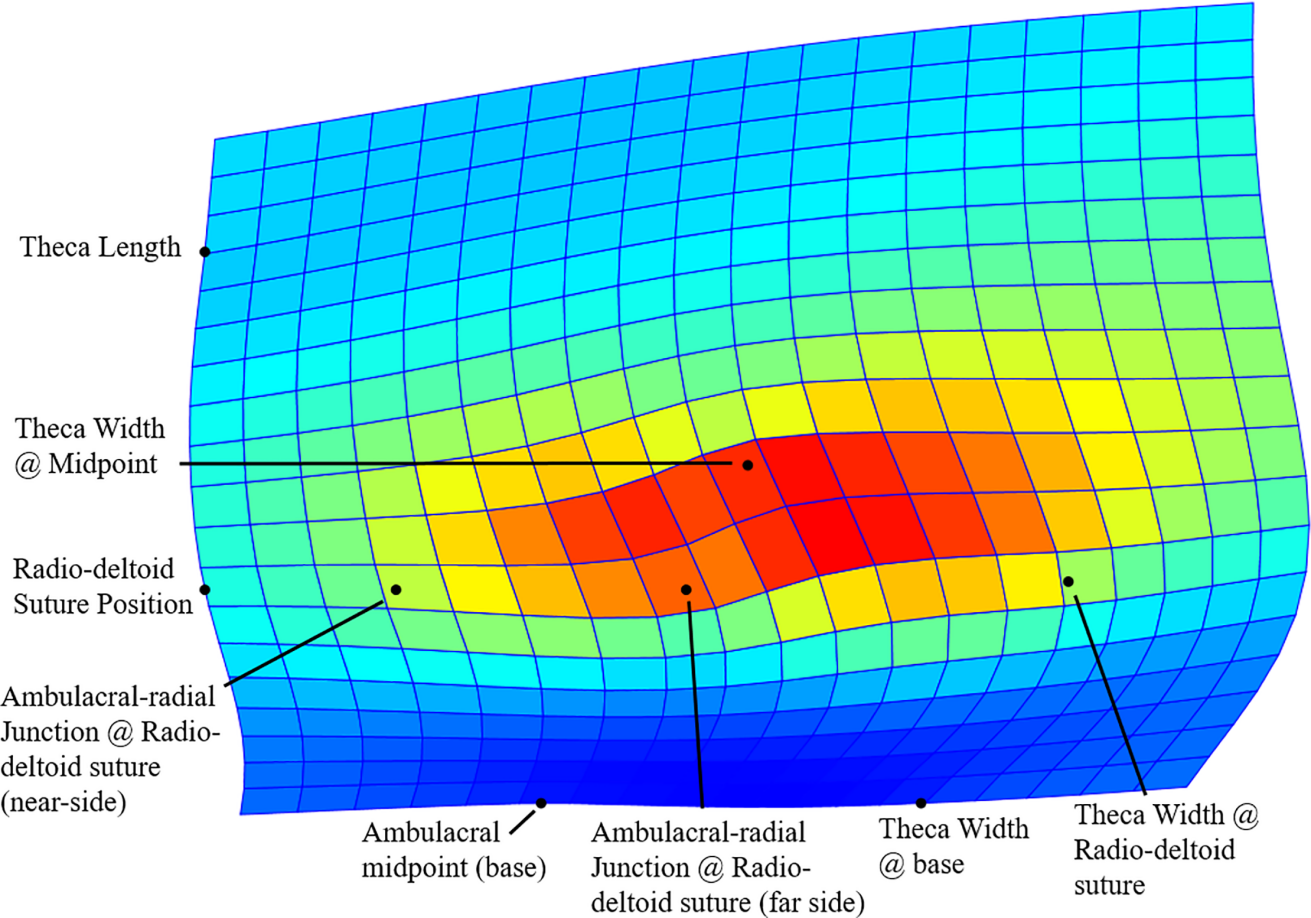
Thin-plate spline analysis results for converted landmark data. Blue = little change, Red = high degree of change. Y-axis corresponds to bilateral axis of blastoid species centered on anal pore. Red region (most alteration between the two compared species) corresponds to upper theca ambulacral region.

Following measurement, *t*-tests performed demonstrated these measurements were significantly different (Table 1). These average measurements for the two species were converted into landmark (x, y) data. These data were loaded into the statistical program PAST [18], where thin-plate splines were calculated (Fig 2). Two-dimensional thin-plate spline analysis utilizes average grid points on a Cartesian plane to calculate change between a set of points [19–20]. The resulting image (spline) produced aids in visualizing differences, if any, which are present between the two input species. More significant differences, or warps, are displayed in red, and the least change is displayed in blue.

## Results

T-test results of *D*. *batheri* and *D*. *delta* average morphological measurements demonstrates significant difference exists between the two closely related species among all measured parameters (Table 1). The largest variances in the measured data were in the upper half of the theca, and were most influential on the dimensions of the ambulacra. Thin-plate spline analysis demonstrates that the changes in upper ambulacra exposure are very evident (red grid sections, Fig 2). The regions of the ambulacra most demonstrably different from each other are primarily concerned with mid-theca widths, such as radio-deltoid suture width, ambulacral width, and average theca width.

## Discussion

The ambulacral region in blastoids, as with many echinoderms, serves two purposes: 1) the primary use is to gather and move food to the oral openings; and 2) to protect the underlying hydrospires, used for respiration and reproduction [21–22]. Differences within the ambulacral region not affecting the oral region or lower theca suggest that the closely related *D*. *batheri* and *D*. *delta* have undergone feeding-based niche partitioning, eliciting alteration to the ambulacra themselves, which would directly affect size and type of food particles that the brachioles could entrain. Feeding niche partitioning is hypothesized based on: inflation of structures near the midline, which is important to the gathering of food; the lack of great differences along the anal region and upper ambulacra, where eggs develop and are released, and would be most greatly influenced if reproduction were the cause of the observed differences between the two species; and the lack of overall change in abundance of either species, suggesting that the differences within the ambulacral region did not lead to an increase in one species over the other, allowing it to proliferate. Rather, both species live within the same units at apparently the same time, along with many other *Deltoblastus* species, suggesting a stable coexistence.

It has been suggested that the close geographic affinity of closely related organisms can be the result of environmental filtering, and, when coupled with flexible phenotypes, genotypic locking. Past workers on *Deltoblastus* species recognized many of them not as separate taxa, but as variants of established species [2–4]. The high diversity of *Deltoblastus* within a closed geographic region could then be the result of environmental filtering and not niche partitioning. Recent work on *Deltoblastus* species, however, has not supported reverting these species to subspecies [1; 5], and therefore niche partitioning is the better-supported hypothesis.

## Conclusions

Study of *D*. *batheri* and *D*. *delta*, two closely related species, reveals differences in the ambulacra region to be the greatest difference morphologically between them, despite an overall significant difference among all traits measured. These differences on contemporaneous species are interpreted as follows:

- *D*. *batheri* and *D*. *delta* are two closely related species occupying the same niche space and geographic area during the same period.
- Being concurrent geographically would place these species in direct competition.
- Thin-plate spline analysis shows notable differences in the size and placement of the ambulacra, but limited differences among other parts of the theca.
- Ambulacral differentiation would impact food gathering capability, and the large differences observed signify food niche differentiation, lessening competition.
- Other options, such as reproductive strategy adjustments or environmental adaptations, are not supported by this study, as these species continue living proximally, and these differences to the ambulacra are not otherwise reflected in competitive advantages or disadvantages.

## Supporting information

S1 Appendix Table of specimens used for this study with repository information for NMUK. (DOCX)

S2 Appendix Landmark data of *D*. *batheri* and *D*. *delta* used for generating warps. (XLSX)
