# Supplementary material for: Niche partitioning as a mechanism for locally high species diversity within a geographically limited genus of blastoid
Source: PLoS One. 2018 May 16;13(5):e0197512. doi: 10.1371/journal.pone.0197512 (PMC5955570; doi:10.1371/journal.pone.0197512)
Supplement: S1 Appendix — (DOCX) [file pone.0197512.s001.docx]

| Genus | Species | ID # | Series | Locale | Province | Drawer |
| --- | --- | --- | --- | --- | --- | --- |
| *Deltoblastus* | *batheri* | e59427 | ? | Some Peh | ? | 60A33 |
| *Deltoblastus* | *batheri* | e59409 | ? | Noil Fatu | ? | 60A33 |
| *Deltoblastus* | *batheri* | e60032 | Sonnebait | ? | ? | 60A33 |
| *Deltoblastus* | *batheri* | e30994 | Sonnebait | Toenioen Eno | Amanoebang | 60A33 |
| *Deltoblastus* | *batheri* | e59035 | Sonnebait | Basleo | Amanoebang | 60A33 |
| *Deltoblastus* | *batheri* | e30992 | Sonnebait | Toenioen Eno | Amanoebang | 60A33 |
| *Deltoblastus* | *batheri* | e59975 | Sonnebait | Toenioen Eno | Amanoebang | 60A33 |
| *Deltoblastus* | *batheri* | e59024 | Sonnebait | Kiomoko | Amanoebang | 60A33 |
| *Deltoblastus* | *batheri* | e30266 | ? | Basleo | Amanoebang | 60A33 |
| *Deltoblastus* | *batheri* | e59073 | Sonnebait | Basleo | Amanoebang | 60A33 |
| *Deltoblastus* | *batheri* | e59038 | Sonnebait | Basleo | Amanoebang | 60A33 |
| *Deltoblastus* | *batheri* | e59698 | Sonnebait | Neoetpantoekak | Amanoebang | 60A33 |
| *Deltoblastus* | *batheri* | e59037 | Sonnebait | Basleo | Amanoebang | 60A33 |
| *Deltoblastus* | *batheri* | e59428 | ? | Some Peh | ? | 60A33 |
| *Deltoblastus* | *batheri* | e59068 | Sonnebait | Basleo | Amanoebang | 60A33 |
| *Deltoblastus* | *batheri* | e59426 | ? | Some Peh | ? | 60A33 |
| *Deltoblastus* | *batheri* | e59026 | Sonnebait | Kiomoko | Amanoebang | 60A33 |
| *Deltoblastus* | *batheri* | e59064 | Sonnebait | Basleo | Amanoebang | 60A33 |
| *Deltoblastus* | *batheri* | e59071 | Sonnebait | Basleo | Amanoebang | 60A33 |
| *Deltoblastus* | *batheri* | e59424 | ? | Some Peh | ? | 60A33 |
| *Deltoblastus* | *batheri* | e59036 | Sonnebait | Basleo | Amanoebang | 60A33 |
| *Deltoblastus* | *batheri* | e59042 | Sonnebait | Basleo | Amanoebang | 60A33 |
| *Deltoblastus* | *batheri* | e59192 | Sonnebait | Soempak | Amanoebang | 60A33 |
| *Deltoblastus* | *batheri* | e59039 | Sonnebait | Basleo | Amanoebang | 60A33 |
| *Deltoblastus* | *batheri* | e59976 | Sonnebait | Toenioen Eno | Amanoebang | 60A33 |
| *Deltoblastus* | *batheri* | e30265 | ? | Basleo | Amanoebang | 60A33 |
| *Deltoblastus* | *batheri* | e59879 | Sonnebait | Kiomoko | Amanoebang | 60A33 |
| *Deltoblastus* | *batheri* | e59028 | Sonnebait | Kiomoko | Amanoebang | 60A33 |
| *Deltoblastus* | *batheri* | e30990 | Sonnebait | Toenioen Eno | Amanoebang | 60A33 |
| *Deltoblastus* | *batheri* | e59041 | Sonnebait | Basleo | Amanoebang | 60A33 |
| *Deltoblastus* | *batheri* | e59040 | Sonnebait | Basleo | Amanoebang | 60A33 |
| *Deltoblastus* | *batheri* | e59049 | Sonnebait | Basleo | Amanoebang | 60A33 |
| *Deltoblastus* | *batheri* | e59057 | Sonnebait | Basleo | Amanoebang | 60A33 |
| *Deltoblastus* | *batheri* | e59191 | Sonnebait | Soempak | Amanoebang | 60A33 |
| *Deltoblastus* | *batheri* | e59050 | Sonnebait | Basleo | Amanoebang | 60A33 |
| *Deltoblastus* | *batheri* | e59043 | Sonnebait | Basleo | Amanoebang | 60A33 |
| *Deltoblastus* | *batheri* | e59054 | Sonnebait | Basleo | Amanoebang | 60A33 |
| *Deltoblastus* | *batheri* | e59046 | Sonnebait | Basleo | Amanoebang | 60A33 |
| *Deltoblastus* | *batheri* | e59074 | Sonnebait | Basleo | Amanoebang | 60A33 |
| *Deltoblastus* | *batheri* | e59700 | Sonnebait | Neoetpantoekak | Amanoebang | 60A33 |
| *Deltoblastus* | *batheri* | e59977 | Sonnebait | Toenioen Eno | Amanoebang | 60A33 |
| *Deltoblastus* | *batheri* | e30469 | ? | Basleo | ? | 60A33 |
| *Deltoblastus* | *batheri* | e30264 | ? | Basleo | Amanoebang | 60A33 |
| *Deltoblastus* | *batheri* | e59979 | Sonnebait | Toenioen Eno | Amanoebang | 60A33 |
| *Deltoblastus* | *batheri* | e59052 | Sonnebait | Basleo | Amanoebang | 60A33 |
| *Deltoblastus* | *batheri* | e59027 | Sonnebait | Kiomoko | Amanoebang | 60A33 |
| *Deltoblastus* | *batheri* | e59699 | Sonnebait | Neoetpantoekak | Amanoebang | 60A33 |
| *Deltoblastus* | *batheri* | e59056 | Sonnebait | Basleo | Amanoebang | 60A33 |
| *Deltoblastus* | *batheri* | e59066 | Sonnebait | Basleo | Amanoebang | 60A33 |
| *Deltoblastus* | *batheri* | e59029 | Sonnebait | Kiomoko | Amanoebang | 60A33 |
| *Deltoblastus* | *batheri* | e59190 | Sonnebait | Soempak | Amanoebang | 60A33 |
| *Deltoblastus* | *batheri* | e59065 | Sonnebait | Basleo | Amanoebang | 60A33 |
| *Deltoblastus* | *batheri* | e59044 | Sonnebait | Basleo | Amanoebang | 60A33 |
| *Deltoblastus* | *batheri* | e59079 | Sonnebait | Basleo | Amanoebang | 60A33 |
| *Deltoblastus* | *batheri* | e59051 | Sonnebait | Basleo | Amanoebang | 60A33 |
| *Deltoblastus* | *batheri* | e59069 | Sonnebait | Basleo | Amanoebang | 60A33 |
| *Deltoblastus* | *batheri* | e59031 | Sonnebait | Kiomoko | Amanoebang | 60A33 |
| *Deltoblastus* | *batheri* | e59978 | Sonnebait | Toenioen Eno | Amanoebang | 60A33 |
| *Deltoblastus* | *batheri* | e59061 | Sonnebait | Basleo | Amanoebang | 60A33 |
| *Deltoblastus* | *batheri* | e59045 | Sonnebait | Basleo | Amanoebang | 60A33 |
| *Deltoblastus* | *batheri* | e59055 | Sonnebait | Basleo | Amanoebang | 60A33 |
| *Deltoblastus* | *batheri* | e59047 | Sonnebait | Basleo | Amanoebang | 60A33 |
| *Deltoblastus* | *batheri* | e59053 | Sonnebait | Basleo | Amanoebang | 60A33 |
| *Deltoblastus* | *batheri* | e30991 | Sonnebait | Toenioen Eno | Amanoebang | 60A33 |
| *Deltoblastus* | *batheri* | e59080 | Sonnebait | Basleo | Amanoebang | 60A33 |
| *Deltoblastus* | *batheri* | e59980 | Sonnebait | Toenioen Eno | Amanoebang | 60A33 |
| *Deltoblastus* | *batheri* | e59048 | Sonnebait | Basleo | Amanoebang | 60A33 |
| *Deltoblastus* | *batheri* | e60016 | Sonnebait | Toenioen Eno | Amanoebang | 60A33 |
| *Deltoblastus* | *delta* | e59742 | Sonnebait | Basleo | Amanoebang | 60A34 |
| *Deltoblastus* | *delta* | e59201 | Sonnebait | Neoetpantoekak | Amanoebang | 60A34 |
| *Deltoblastus* | *delta* | e59732 | Sonnebait | Basleo | Amanoebang | 60A34 |
| *Deltoblastus* | *delta* | e59182 | Sonnebait | Soempak | Amanoebang | 60A34 |
| *Deltoblastus* | *delta* | e30752 | Sonnebait | Faoet Ao | Amanoebang | 60A33 |
| *Deltoblastus* | *delta* | e59734 | Sonnebait | Basleo | Amanoebang | 60A34 |
| *Deltoblastus* | *delta* | e59736 | Sonnebait | Basleo | Amanoebang | 60A34 |
| *Deltoblastus* | *delta* | e59809 | Sonnebait | Soem Peh | Amanoebang | 60A34 |
| *Deltoblastus* | *delta* | e30962 | Sonnebait | Kioemoko | Amanoebang | 60A34 |
| *Deltoblastus* | *delta* | e59185 | Sonnebait | Soempak | Amanoebang | 60A34 |
| *Deltoblastus* | *delta* | e59184 | Sonnebait | Soempak | Amanoebang | 60A34 |
| *Deltoblastus* | *delta* | e30969 | Sonnebait | Kioemoko | Amanoebang | 60A34 |
| *Deltoblastus* | *delta* | e59683 | Sonnebait | Neoetpantoekak | Amanoebang | 60A34 |
| *Deltoblastus* | *delta* | e30968 | Sonnebait | Kioemoko | Amanoebang | 60A34 |
| *Deltoblastus* | *delta* | e30907 | Sonnebait | Basleo | Amanoebang | 60A33 |
| *Deltoblastus* | *delta* | e59948 | Sonnebait | Toenioen Eno | Amanoebang | 60A35 |
| *Deltoblastus* | *delta* | e30743 | Sonnebait | Faoet Ao | Amanoebang | 60A33 |
| *Deltoblastus* | *delta* | e59179 | Sonnebait | Soempak | Amanoebang | 60A34 |
| *Deltoblastus* | *delta* | e59737 | Sonnebait | Basleo | Amanoebang | 60A34 |
| *Deltoblastus* | *delta* | e30963 | Sonnebait | Kioemoko | Amanoebang | 60A34 |
| *Deltoblastus* | *delta* | e30745 | Sonnebait | Faoet Ao | Amanoebang | 60A33 |
| *Deltoblastus* | *delta* | e59851 | Sonnebait | Kioemoko | Amanoebang | 60A34 |
| *Deltoblastus* | *delta* | e30934 | Sonnebait | Basleo | Amanoebang | 60A33 |
| *Deltoblastus* | *delta* | e30912 | Sonnebait | Basleo | Amanoebang | 60A33 |
| *Deltoblastus* | *delta* | e59729 | Sonnebait | Basleo | Amanoebang | 60A34 |
| *Deltoblastus* | *delta* | e59171 | Sonnebait | Soempak | Amanoebang | 60A34 |
| *Deltoblastus* | *delta* | e30753 | Sonnebait | Faoet Ao | Amanoebang | 60A33 |
| *Deltoblastus* | *delta* | e60014 (25) | Sonnebait | Toenioen Eno | Amanoebang | 60A34 |
| *Deltoblastus* | *delta* | e59187 | Sonnebait | Soempak | Amanoebang | 60A34 |
| *Deltoblastus* | *delta* | e59690 | Sonnebait | Neoetpantoekak | Amanoebang | 60A34 |
| *Deltoblastus* | *delta* | e30761 | Sonnebait | Faoet Ao | Amanoebang | 60A33 |
| *Deltoblastus* | *delta* | e30964 | Sonnebait | Kioemoko | Amanoebang | 60A34 |
| *Deltoblastus* | *delta* | e59808 | Sonnebait | Soem Peh | Amanoebang | 60A34 |
| *Deltoblastus* | *delta* | e60014 (25) | Sonnebait | Toenioen Eno | Amanoebang | 60A34 |
| *Deltoblastus* | *delta* | e59860 | Sonnebait | Kioemoko | Amanoebang | 60A34 |
| *Deltoblastus* | *delta* | e60014 (25) | Sonnebait | Toenioen Eno | Amanoebang | 60A34 |
| *Deltoblastus* | *delta* | e30913 | Sonnebait | Basleo | Amanoebang | 60A33 |
| *Deltoblastus* | *delta* | e59162 | Sonnebait | Soempak | Amanoebang | 60A34 |
| *Deltoblastus* | *delta* | e30736 | Sonnebait | Faoet Ao | Amanoebang | 60A33 |
| *Deltoblastus* | *delta* | e59807 | Sonnebait | Soem Peh | Amanoebang | 60A34 |
| *Deltoblastus* | *delta* | e59801 | Sonnebait | Soem Peh | Amanoebang | 60A34 |
| *Deltoblastus* | *delta* | e59950 | Sonnebait | Toenioen Eno | Amanoebang | 60A35 |
| *Deltoblastus* | *delta* | e59949 | Sonnebait | Toenioen Eno | Amanoebang | 60A35 |
| *Deltoblastus* | *delta* | e60014 (25) | Sonnebait | Toenioen Eno | Amanoebang | 60A34 |
| *Deltoblastus* | *delta* | e59165 | Sonnebait | Soempak | Amanoebang | 60A34 |
| *Deltoblastus* | *delta* | e59181 | Sonnebait | Soempak | Amanoebang | 60A34 |
| *Deltoblastus* | *delta* | e59692 | Sonnebait | Neoetpantoekak | Amanoebang | 60A34 |
| *Deltoblastus* | *delta* | e30767 | Sonnebait | Faoet Ao | Amanoebang | 60A33 |
| *Deltoblastus* | *delta* | e59957 | Sonnebait | Toenioen Eno | Amanoebang | 60A35 |
| *Deltoblastus* | *delta* | e59740 | Sonnebait | Basleo | Amanoebang | 60A34 |
| *Deltoblastus* | *delta* | e59848 | Sonnebait | Kioemoko | Amanoebang | 60A34 |
| *Deltoblastus* | *delta* | e59895 | Sonnebait | Faoet Ao | Amanoebang | 60A33 |
| *Deltoblastus* | *delta* | e59891 | Sonnebait | Faoet Ao | Amanoebang | 60A33 |
| *Deltoblastus* | *delta* | e59156 | Sonnebait | Soempak | Amanoebang | 60A34 |
| *Deltoblastus* | *delta* | e59234 | Sonnebait | Neoetpantoekak | Amanoebang | 60A34 |
| *Deltoblastus* | *delta* | e30920 | Sonnebait | Basleo | Amanoebang | 60A34 |
| *Deltoblastus* | *delta* | e59863 | Sonnebait | Kioemoko | Amanoebang | 60A34 |
| *Deltoblastus* | *delta* | e30967 | Sonnebait | Kioemoko | Amanoebang | 60A34 |
| *Deltoblastus* | *delta* | e30757 | Sonnebait | Faoet Ao | Amanoebang | 60A33 |
| *Deltoblastus* | *delta* | e30732 | Sonnebait | Faoet Ao | Amanoebang | 60A33 |
| *Deltoblastus* | *delta* | e30940 | Sonnebait | Basleo | Amanoebang | 60A33 |
| *Deltoblastus* | *delta* | e60014 (25) | Sonnebait | Toenioen Eno | Amanoebang | 60A34 |
| *Deltoblastus* | *delta* | e30922 | Sonnebait | Basleo | Amanoebang | 60A33 |
| *Deltoblastus* | *delta* | e59176 | Sonnebait | Soempak | Amanoebang | 60A34 |
| *Deltoblastus* | *delta* | e30759 | Sonnebait | Faoet Ao | Amanoebang | 60A33 |
| *Deltoblastus* | *delta* | e30953 | Sonnebait | Kioemoko | Amanoebang | 60A34 |
| *Deltoblastus* | *delta* | e59167 | Sonnebait | Soempak | Amanoebang | 60A34 |
| *Deltoblastus* | *delta* | e30741 | Sonnebait | Faoet Ao | Amanoebang | 60A33 |
| *Deltoblastus* | *delta* | e59195 | Sonnebait | Neoetpantoekak | Amanoebang | 60A33 |
| *Deltoblastus* | *delta* | e59811 | Sonnebait | Soem Peh | Amanoebang | 60A34 |
| *Deltoblastus* | *delta* | e30923 | Sonnebait | Basleo | Amanoebang | 60A33 |
| *Deltoblastus* | *delta* | e60014 (25) | Sonnebait | Toenioen Eno | Amanoebang | 60A34 |
| *Deltoblastus* | *delta* | e59222 | Sonnebait | Neoetpantoekak | Amanoebang | 60A34 |
| *Deltoblastus* | *delta* | e59953 | Sonnebait | Toenioen Eno | Amanoebang | 60A35 |
| *Deltoblastus* | *delta* | e30756 | Sonnebait | Faoet Ao | Amanoebang | 60A33 |
| *Deltoblastus* | *delta* | e59951 | Sonnebait | Toenioen Eno | Amanoebang | 60A35 |
| *Deltoblastus* | *delta* | e59155 | Sonnebait | Soempak | Amanoebang | 60A34 |
| *Deltoblastus* | *delta* | e59148 | Sonnebait | Soempak | Amanoebang | 60A34 |
| *Deltoblastus* | *delta* | e30751 | Sonnebait | Faoet Ao | Amanoebang | 60A33 |
| *Deltoblastus* | *delta* | e60025 (40) | Sonnebait | Faoet Ao | Amanoebang | 60A33 |
| *Deltoblastus* | *delta* | e59151 | Sonnebait | Soempak | Amanoebang | 60A34 |
| *Deltoblastus* | *delta* | e60014 (25) | Sonnebait | Toenioen Eno | Amanoebang | 60A34 |
| *Deltoblastus* | *delta* | e59847 | Sonnebait | Kioemoko | Amanoebang | 60A34 |
| *Deltoblastus* | *delta* | e60014 (25) | Sonnebait | Toenioen Eno | Amanoebang | 60A34 |
| *Deltoblastus* | *delta* | e59852 | Sonnebait | Kioemoko | Amanoebang | 60A34 |
| *Deltoblastus* | *delta* | e59894 | Sonnebait | Faoet Ao | Amanoebang | 60A33 |
| *Deltoblastus* | *delta* | e59238 | Sonnebait | Neoetpantoekak | Amanoebang | 60A34 |
| *Deltoblastus* | *delta* | e59173 | Sonnebait | Soempak | Amanoebang | 60A34 |
| *Deltoblastus* | *delta* | e59158 | Sonnebait | Soempak | Amanoebang | 60A34 |
| *Deltoblastus* | *delta* | e59170 | Sonnebait | Soempak | Amanoebang | 60A34 |
| *Deltoblastus* | *delta* | e59726 | Sonnebait | Basleo | Amanoebang | 60A34 |
| *Deltoblastus* | *delta* | e60025 (40) | Sonnebait | Faoet Ao | Amanoebang | 60A33 |
| *Deltoblastus* | *delta* | e60025 (40) | Sonnebait | Faoet Ao | Amanoebang | 60A33 |
| *Deltoblastus* | *delta* | e59216 | Sonnebait | Neoetpantoekak | Amanoebang | 60A34 |
| *Deltoblastus* | *delta* | e59846 | Sonnebait | Kioemoko | Amanoebang | 60A34 |
| *Deltoblastus* | *delta* | e30988 | Sonnebait | Kiomoko | Amanoebang | 60A33 |
| *Deltoblastus* | *delta* | e59964 | Sonnebait | Toenioen Eno | Amanoebang | 60A35 |
| *Deltoblastus* | *delta* | e59688 | Sonnebait | Neoetpantoekak | Amanoebang | 60A34 |
| *Deltoblastus* | *delta* | e59850 | Sonnebait | Kioemoko | Amanoebang | 60A34 |
| *Deltoblastus* | *delta* | e59892 | Sonnebait | Faoet Ao | Amanoebang | 60A33 |
| *Deltoblastus* | *delta* | e60025 (40) | Sonnebait | Faoet Ao | Amanoebang | 60A33 |
| *Deltoblastus* | *delta* | e59810 | Sonnebait | Soem Peh | Amanoebang | 60A34 |
| *Deltoblastus* | *delta* | e30750 | Sonnebait | Faoet Ao | Amanoebang | 60A33 |
| *Deltoblastus* | *delta* | e30965 | Sonnebait | Kioemoko | Amanoebang | 60A34 |
| *Deltoblastus* | *delta* | e59166 | Sonnebait | Soempak | Amanoebang | 60A34 |
| *Deltoblastus* | *delta* | e60025 (40) | Sonnebait | Faoet Ao | Amanoebang | 60A33 |
| *Deltoblastus* | *delta* | e59168 | Sonnebait | Soempak | Amanoebang | 60A34 |
| *Deltoblastus* | *delta* | e59153 | Sonnebait | Soempak | Amanoebang | 60A34 |
| *Deltoblastus* | *delta* | e59684 | Sonnebait | Neoetpantoekak | Amanoebang | 60A34 |
| *Deltoblastus* | *delta* | e59741 | Sonnebait | Basleo | Amanoebang | 60A34 |
| *Deltoblastus* | *delta* | e59180 | Sonnebait | Soempak | Amanoebang | 60A34 |
| *Deltoblastus* | *delta* | e59803 | Sonnebait | Soem Peh | Amanoebang | 60A34 |
| *Deltoblastus* | *delta* | e60025 (40) | Sonnebait | Faoet Ao | Amanoebang | 60A33 |
| *Deltoblastus* | *delta* | e59799 | Sonnebait | Soem Peh | Amanoebang | 60A34 |
| *Deltoblastus* | *delta* | e30936 | Sonnebait | Basleo | Amanoebang | 60A33 |
| *Deltoblastus* | *delta* | e59857 | Sonnebait | Kioemoko | Amanoebang | 60A34 |
| *Deltoblastus* | *delta* | e30764 | Sonnebait | Faoet Ao | Amanoebang | 60A33 |
| *Deltoblastus* | *delta* | e59959 | Sonnebait | Toenioen Eno | Amanoebang | 60A35 |
| *Deltoblastus* | *delta* | e59738 | Sonnebait | Basleo | Amanoebang | 60A34 |
| *Deltoblastus* | *delta* | e59794 | Sonnebait | Soem Peh | Amanoebang | 60A34 |
| *Deltoblastus* | *delta* | e59169 | Sonnebait | Soempak | Amanoebang | 60A34 |
| *Deltoblastus* | *delta* | e30937 | Sonnebait | Basleo | Amanoebang | 60A33 |
| *Deltoblastus* | *delta* | e30763 | Sonnebait | Faoet Ao | Amanoebang | 60A33 |
| *Deltoblastus* | *delta* | e59853 | Sonnebait | Kioemoko | Amanoebang | 60A34 |
| *Deltoblastus* | *delta* | e59952 | Sonnebait | Toenioen Eno | Amanoebang | 60A35 |
| *Deltoblastus* | *delta* | e59175 | Sonnebait | Soempak | Amanoebang | 60A34 |
| *Deltoblastus* | *delta* | e30762 | Sonnebait | Faoet Ao | Amanoebang | 60A33 |
| *Deltoblastus* | *delta* | e60014 (25) | Sonnebait | Toenioen Eno | Amanoebang | 60A34 |
| *Deltoblastus* | *delta* | e30950 | Sonnebait | Kioemoko | Amanoebang | 60A34 |
| *Deltoblastus* | *delta* | e30955 | Sonnebait | Kioemoko | Amanoebang | 60A34 |
| *Deltoblastus* | *delta* | e60025 (40) | Sonnebait | Faoet Ao | Amanoebang | 60A33 |
| *Deltoblastus* | *delta* | e59731 | Sonnebait | Basleo | Amanoebang | 60A34 |
| *Deltoblastus* | *delta* | e59691 | Sonnebait | Neoetpantoekak | Amanoebang | 60A34 |
| *Deltoblastus* | *delta* | e59687 | Sonnebait | Neoetpantoekak | Amanoebang | 60A34 |
| *Deltoblastus* | *delta* | e30740 | Sonnebait | Faoet Ao | Amanoebang | 60A33 |
| *Deltoblastus* | *delta* | e59792 | Sonnebait | Soem Peh | Amanoebang | 60A34 |
| *Deltoblastus* | *delta* | e30725 | Sonnebait | Faoet Ao | Amanoebang | 60A33 |
| *Deltoblastus* | *delta* | e59961 | Sonnebait | Toenioen Eno | Amanoebang | 60A35 |
| *Deltoblastus* | *delta* | e60025 (40) | Sonnebait | Faoet Ao | Amanoebang | 60A33 |
| *Deltoblastus* | *delta* | e59739 | Sonnebait | Basleo | Amanoebang | 60A34 |
| *Deltoblastus* | *delta* | e30989 | Sonnebait | Kiomoko | Amanoebang | 60A33 |
| *Deltoblastus* | *delta* | e59856 | Sonnebait | Kioemoko | Amanoebang | 60A34 |
| *Deltoblastus* | *delta* | e59804 | Sonnebait | Soem Peh | Amanoebang | 60A34 |
| *Deltoblastus* | *delta* | e60014 (25) | Sonnebait | Toenioen Eno | Amanoebang | 60A34 |
| *Deltoblastus* | *delta* | e30724 | Sonnebait | Faoet Ao | Amanoebang | 60A33 |
| *Deltoblastus* | *delta* | e59945 | Sonnebait | Toenioen Eno | Amanoebang | 60A35 |
| *Deltoblastus* | *delta* | e30766 | Sonnebait | Faoet Ao | Amanoebang | 60A33 |
| *Deltoblastus* | *delta* | e30742 | Sonnebait | Faoet Ao | Amanoebang | 60A33 |
| *Deltoblastus* | *delta* | e30746 | Sonnebait | Faoet Ao | Amanoebang | 60A33 |
| *Deltoblastus* | *delta* | e30755 | Sonnebait | Faoet Ao | Amanoebang | 60A33 |
| *Deltoblastus* | *delta* | e59154 | Sonnebait | Soempak | Amanoebang | 60A34 |
| *Deltoblastus* | *delta* | e30966 | Sonnebait | Kioemoko | Amanoebang | 60A34 |
| *Deltoblastus* | *delta* | e59806 | Sonnebait | Soem Peh | Amanoebang | 60A34 |
| *Deltoblastus* | *delta* | e60025 (40) | Sonnebait | Faoet Ao | Amanoebang | 60A33 |
| *Deltoblastus* | *delta* | e60025 (40) | Sonnebait | Faoet Ao | Amanoebang | 60A33 |
| *Deltoblastus* | *delta* | e30979 | Sonnebait | Kiomoko | Amanoebang | 60A33 |
| *Deltoblastus* | *delta* | e30758 | Sonnebait | Faoet Ao | Amanoebang | 60A33 |
| *Deltoblastus* | *delta* | e59800 | Sonnebait | Soem Peh | Amanoebang | 60A34 |
| *Deltoblastus* | *delta* | e59204 | Sonnebait | Neoetpantoekak | Amanoebang | 60A34 |
| *Deltoblastus* | *delta* | e59225 | Sonnebait | Neoetpantoekak | Amanoebang | 60A34 |
| *Deltoblastus* | *delta* | e59900 | Sonnebait | Faoet Ao | Amanoebang | 60A33 |
| *Deltoblastus* | *delta* | e59172 | Sonnebait | Soempak | Amanoebang | 60A34 |
| *Deltoblastus* | *delta* | e60025 (40) | Sonnebait | Faoet Ao | Amanoebang | 60A33 |
| *Deltoblastus* | *delta* | e59730 | Sonnebait | Basleo | Amanoebang | 60A34 |
| *Deltoblastus* | *delta* | e59733 | Sonnebait | Basleo | Amanoebang | 60A34 |
| *Deltoblastus* | *delta* | e59241 | Sonnebait | Neoetpantoekak | Amanoebang | 60A34 |
| *Deltoblastus* | *delta* | e60014 (25) | Sonnebait | Toenioen Eno | Amanoebang | 60A34 |
| *Deltoblastus* | *delta* | e30958 | Sonnebait | Kioemoko | Amanoebang | 60A34 |
| *Deltoblastus* | *delta* | e60025 (40) | Sonnebait | Faoet Ao | Amanoebang | 60A33 |
| *Deltoblastus* | *delta* | e59963 | Sonnebait | Toenioen Eno | Amanoebang | 60A35 |
| *Deltoblastus* | *delta* | e59152 | Sonnebait | Soempak | Amanoebang | 60A34 |
| *Deltoblastus* | *delta* | e59965 | Sonnebait | Toenioen Eno | Amanoebang | 60A35 |
| *Deltoblastus* | *delta* | e30748 | Sonnebait | Faoet Ao | Amanoebang | 60A33 |
| *Deltoblastus* | *delta* | e59956 | Sonnebait | Toenioen Eno | Amanoebang | 60A35 |
| *Deltoblastus* | *delta* | e60014 (25) | Sonnebait | Toenioen Eno | Amanoebang | 60A34 |
| *Deltoblastus* | *delta* | e59143 | Sonnebait | Soempak | Amanoebang | 60A34 |
| *Deltoblastus* | *delta* | e60025 (40) | Sonnebait | Faoet Ao | Amanoebang | 60A33 |
| *Deltoblastus* | *delta* | e60025 (40) | Sonnebait | Faoet Ao | Amanoebang | 60A33 |
| *Deltoblastus* | *delta* | e59795 | Sonnebait | Soem Peh | Amanoebang | 60A34 |
| *Deltoblastus* | *delta* | e60025 (40) | Sonnebait | Faoet Ao | Amanoebang | 60A33 |
| *Deltoblastus* | *delta* | e59174 | Sonnebait | Soempak | Amanoebang | 60A34 |
| *Deltoblastus* | *delta* | e30960 | Sonnebait | Kioemoko | Amanoebang | 60A34 |
| *Deltoblastus* | *delta* | e59157 (2) | Sonnebait | Soem Peh | Amanoebang | 60A35 |
| *Deltoblastus* | *delta* | e59227 | Sonnebait | Neoetpantoekak | Amanoebang | 60A34 |
| *Deltoblastus* | *delta* | e59183 | Sonnebait | Soempak | Amanoebang | 60A34 |
| *Deltoblastus* | *delta* | e30931 | Sonnebait | Basleo | Amanoebang | 60A33 |
| *Deltoblastus* | *delta* | e30980 | Sonnebait | Kiomoko | Amanoebang | 60A33 |
| *Deltoblastus* | *delta* | e59725 | Sonnebait | Basleo | Amanoebang | 60A34 |
| *Deltoblastus* | *delta* | e60014 (25) | Sonnebait | Toenioen Eno | Amanoebang | 60A34 |
| *Deltoblastus* | *delta* | e59197 | Sonnebait | Neoetpantoekak | Amanoebang | 60A33 |
| *Deltoblastus* | *delta* | e59735 | Sonnebait | Basleo | Amanoebang | 60A34 |
| *Deltoblastus* | *delta* | e59861 | Sonnebait | Kioemoko | Amanoebang | 60A34 |
| *Deltoblastus* | *delta* | e60025 (40) | Sonnebait | Faoet Ao | Amanoebang | 60A33 |
| *Deltoblastus* | *delta* | e59802 | Sonnebait | Soem Peh | Amanoebang | 60A34 |
| *Deltoblastus* | *delta* | e30952 | Sonnebait | Kioemoko | Amanoebang | 60A34 |
| *Deltoblastus* | *delta* | e60025 (40) | Sonnebait | Faoet Ao | Amanoebang | 60A33 |
| *Deltoblastus* | *delta* | e30754 | Sonnebait | Faoet Ao | Amanoebang | 60A33 |
| *Deltoblastus* | *delta* | e59146 | Sonnebait | Soempak | Amanoebang | 60A34 |
| *Deltoblastus* | *delta* | e59232 | Sonnebait | Neoetpantoekak | Amanoebang | 60A34 |
| *Deltoblastus* | *delta* | e60025 (40) | Sonnebait | Faoet Ao | Amanoebang | 60A33 |
| *Deltoblastus* | *delta* | e60025 (40) | Sonnebait | Faoet Ao | Amanoebang | 60A33 |
| *Deltoblastus* | *delta* | e59160 | Sonnebait | Soempak | Amanoebang | 60A34 |
| *Deltoblastus* | *delta* | e59219 | Sonnebait | Neoetpantoekak | Amanoebang | 60A34 |
| *Deltoblastus* | *delta* | e59157 (2) | Sonnebait | Soem Peh | Amanoebang | 60A34 |
| *Deltoblastus* | *delta* | e30911 | Sonnebait | Basleo | Amanoebang | 60A33 |
| *Deltoblastus* | *delta* | e59798 | Sonnebait | Soem Peh | Amanoebang | 60A34 |
| *Deltoblastus* | *delta* | e59207 | Sonnebait | Neoetpantoekak | Amanoebang | 60A34 |
| *Deltoblastus* | *delta* | e59960 | Sonnebait | Toenioen Eno | Amanoebang | 60A35 |
| *Deltoblastus* | *delta* | e59962 | Sonnebait | Toenioen Eno | Amanoebang | 60A35 |
| *Deltoblastus* | *delta* | e59214 | Sonnebait | Neoetpantoekak | Amanoebang | 60A34 |
| *Deltoblastus* | *delta* | e59231 | Sonnebait | Neoetpantoekak | Amanoebang | 60A34 |
| *Deltoblastus* | *delta* | e59236 | Sonnebait | Neoetpantoekak | Amanoebang | 60A34 |
| *Deltoblastus* | *delta* | e59164 | Sonnebait | Soempak | Amanoebang | 60A34 |
| *Deltoblastus* | *delta* | e30728 | Sonnebait | Faoet Ao | Amanoebang | 60A33 |
| *Deltoblastus* | *delta* | e30957 | Sonnebait | Kioemoko | Amanoebang | 60A34 |
| *Deltoblastus* | *delta* | e30723 | Sonnebait | Faoet Ao | Amanoebang | 60A33 |
| *Deltoblastus* | *delta* | e30739 | Sonnebait | Faoet Ao | Amanoebang | 60A33 |
| *Deltoblastus* | *delta* | e30986 | Sonnebait | Kiomoko | Amanoebang | 60A33 |
| *Deltoblastus* | *delta* | e30951 | Sonnebait | Kioemoko | Amanoebang | 60A34 |
| *Deltoblastus* | *delta* | e59898 | Sonnebait | Faoet Ao | Amanoebang | 60A33 |
| *Deltoblastus* | *delta* | e30961 | Sonnebait | Kioemoko | Amanoebang | 60A34 |
| *Deltoblastus* | *delta* | e59147 | Sonnebait | Soempak | Amanoebang | 60A34 |
| *Deltoblastus* | *delta* | e60014 (25) | Sonnebait | Toenioen Eno | Amanoebang | 60A34 |
| *Deltoblastus* | *delta* | e59150 | Sonnebait | Soempak | Amanoebang | 60A34 |
| *Deltoblastus* | *delta* | e30985 | Sonnebait | Kiomoko | Amanoebang | 60A33 |
| *Deltoblastus* | *delta* | e59203 | Sonnebait | Neoetpantoekak | Amanoebang | 60A34 |
| *Deltoblastus* | *delta* | e59159 | Sonnebait | Soempak | Amanoebang | 60A34 |
| *Deltoblastus* | *delta* | e59790 | Sonnebait | Soem Peh | Amanoebang | 60A34 |
| *Deltoblastus* | *delta* | e30731 | Sonnebait | Faoet Ao | Amanoebang | 60A33 |
| *Deltoblastus* | *delta* | e30737 | Sonnebait | Faoet Ao | Amanoebang | 60A33 |
| *Deltoblastus* | *delta* | e59198 | Sonnebait | Neoetpantoekak | Amanoebang | 60A33 |
| *Deltoblastus* | *delta* | e59242 | Sonnebait | Neoetpantoekak | Amanoebang | 60A34 |
| *Deltoblastus* | *delta* | e59200 | Sonnebait | Neoetpantoekak | Amanoebang | 60A34 |
| *Deltoblastus* | *delta* | e60025 (40) | Sonnebait | Faoet Ao | Amanoebang | 60A33 |
| *Deltoblastus* | *delta* | e59805 | Sonnebait | Soem Peh | Amanoebang | 60A34 |
| *Deltoblastus* | *delta* | e30978 | Sonnebait | Kiomoko | Amanoebang | 60A33 |
| *Deltoblastus* | *delta* | e59859 | Sonnebait | Kioemoko | Amanoebang | 60A34 |
| *Deltoblastus* | *delta* | e60014 (25) | Sonnebait | Toenioen Eno | Amanoebang | 60A34 |
| *Deltoblastus* | *delta* | e30987 | Sonnebait | Kiomoko | Amanoebang | 60A33 |
| *Deltoblastus* | *delta* | e30917 | Sonnebait | Basleo | Amanoebang | 60A33 |
| *Deltoblastus* | *delta* | e30909 | Sonnebait | Basleo | Amanoebang | 60A33 |
| *Deltoblastus* | *delta* | e59178 | Sonnebait | Soempak | Amanoebang | 60A34 |
| *Deltoblastus* | *delta* | e59212 | Sonnebait | Neoetpantoekak | Amanoebang | 60A34 |
| *Deltoblastus* | *delta* | e30932 | Sonnebait | Basleo | Amanoebang | 60A33 |
| *Deltoblastus* | *delta* | e59897 | Sonnebait | Faoet Ao | Amanoebang | 60A33 |
| *Deltoblastus* | *delta* | e59862 | Sonnebait | Kioemoko | Amanoebang | 60A34 |
| *Deltoblastus* | *delta* | e59797 | Sonnebait | Soem Peh | Amanoebang | 60A34 |
| *Deltoblastus* | *delta* | e30726 | Sonnebait | Faoet Ao | Amanoebang | 60A33 |
| *Deltoblastus* | *delta* | e30722 | Sonnebait | Faoet Ao | Amanoebang | 60A33 |
| *Deltoblastus* | *delta* | e30904 | Sonnebait | Basleo | Amanoebang | 60A33 |
| *Deltoblastus* | *delta* | e59189 | Sonnebait | Soempak | Amanoebang | 60A34 |
| *Deltoblastus* | *delta* | e59149 | Sonnebait | Soempak | Amanoebang | 60A34 |
| *Deltoblastus* | *delta* | e30959 | Sonnebait | Kioemoko | Amanoebang | 60A34 |
| *Deltoblastus* | *delta* | e30954 | Sonnebait | Kioemoko | Amanoebang | 60A34 |
| *Deltoblastus* | *delta* | e30956 | Sonnebait | Kioemoko | Amanoebang | 60A34 |
| *Deltoblastus* | *delta* | e59858 | Sonnebait | Kioemoko | Amanoebang | 60A34 |
| *Deltoblastus* | *delta* | e30928 | Sonnebait | Basleo | Amanoebang | 60A33 |
| *Deltoblastus* | *delta* | e59849 | Sonnebait | Kioemoko | Amanoebang | 60A34 |
| *Deltoblastus* | *delta* | e59901 | Sonnebait | Faoet Ao | Amanoebang | 60A33 |
| *Deltoblastus* | *delta* | e59958 | Sonnebait | Toenioen Eno | Amanoebang | 60A35 |
| *Deltoblastus* | *delta* | e30718 | Sonnebait | Faoet Ao | Amanoebang | 60A33 |
| *Deltoblastus* | *delta* | e59235 | Sonnebait | Neoetpantoekak | Amanoebang | 60A34 |
| *Deltoblastus* | *delta* | e30719 | Sonnebait | Faoet Ao | Amanoebang | 60A33 |
| *Deltoblastus* | *delta* | e60014 (25) | Sonnebait | Toenioen Eno | Amanoebang | 60A34 |
| *Deltoblastus* | *delta* | e59205 | Sonnebait | Neoetpantoekak | Amanoebang | 60A34 |
| *Deltoblastus* | *delta* | e30730 | Sonnebait | Faoet Ao | Amanoebang | 60A33 |
| *Deltoblastus* | *delta* | e59719 | Sonnebait | Basleo | Amanoebang | 60A34 |
| *Deltoblastus* | *delta* | e59955 | Sonnebait | Toenioen Eno | Amanoebang | 60A35 |
| *Deltoblastus* | *delta* | e30918 | Sonnebait | Basleo | Amanoebang | 60A33 |
| *Deltoblastus* | *delta* | e59718 | Sonnebait | Basleo | Amanoebang | 60A34 |
| *Deltoblastus* | *delta* | e59177 | Sonnebait | Soempak | Amanoebang | 60A34 |
| *Deltoblastus* | *delta* | e59896 | Sonnebait | Faoet Ao | Amanoebang | 60A33 |
| *Deltoblastus* | *delta* | e30946 | Sonnebait | Kioemoko | Amanoebang | 60A34 |
| *Deltoblastus* | *delta* | e59899 | Sonnebait | Faoet Ao | Amanoebang | 60A33 |
| *Deltoblastus* | *delta* | e59209 | Sonnebait | Neoetpantoekak | Amanoebang | 60A34 |
| *Deltoblastus* | *delta* | e60025 (40) | Sonnebait | Faoet Ao | Amanoebang | 60A33 |
| *Deltoblastus* | *delta* | e59796 | Sonnebait | Soem Peh | Amanoebang | 60A34 |
| *Deltoblastus* | *delta* | e59211 | Sonnebait | Neoetpantoekak | Amanoebang | 60A34 |
| *Deltoblastus* | *delta* | e59724 | Sonnebait | Basleo | Amanoebang | 60A34 |
| *Deltoblastus* | *delta* | e30738 | Sonnebait | Faoet Ao | Amanoebang | 60A33 |
| *Deltoblastus* | *delta* | e59161 | Sonnebait | Soempak | Amanoebang | 60A34 |
| *Deltoblastus* | *delta* | e59215 | Sonnebait | Neoetpantoekak | Amanoebang | 60A34 |
| *Deltoblastus* | *delta* | e30916 | Sonnebait | Basleo | Amanoebang | 60A33 |
| *Deltoblastus* | *delta* | e30977 | Sonnebait | Kiomoko | Amanoebang | 60A33 |
| *Deltoblastus* | *delta* | e30975 | Sonnebait | Kiomoko | Amanoebang | 60A33 |
| *Deltoblastus* | *delta* | e30948 | Sonnebait | Kioemoko | Amanoebang | 60A34 |
| *Deltoblastus* | *delta* | e59944 | Sonnebait | Toenioen Eno | Amanoebang | 60A35 |
| *Deltoblastus* | *delta* | e59213 | Sonnebait | Neoetpantoekak | Amanoebang | 60A34 |
| *Deltoblastus* | *delta* | e30721 | Sonnebait | Faoet Ao | Amanoebang | 60A33 |
| *Deltoblastus* | *delta* | e30906 | Sonnebait | Basleo | Amanoebang | 60A33 |
| *Deltoblastus* | *delta* | e59954 | Sonnebait | Toenioen Eno | Amanoebang | 60A35 |
| *Deltoblastus* | *delta* | e59144 | Sonnebait | Soempak | Amanoebang | 60A34 |
| *Deltoblastus* | *delta* | e30908 | Sonnebait | Basleo | Amanoebang | 60A33 |
| *Deltoblastus* | *delta* | e59727 | Sonnebait | Basleo | Amanoebang | 60A34 |
| *Deltoblastus* | *delta* | e59854 | Sonnebait | Kioemoko | Amanoebang | 60A34 |
| *Deltoblastus* | *delta* | e59226 | Sonnebait | Neoetpantoekak | Amanoebang | 60A34 |
| *Deltoblastus* | *delta* | e59145 | Sonnebait | Soempak | Amanoebang | 60A34 |
| *Deltoblastus* | *delta* | e59686 | Sonnebait | Neoetpantoekak | Amanoebang | 60A34 |
| *Deltoblastus* | *delta* | e30947 | Sonnebait | Kioemoko | Amanoebang | 60A34 |
| *Deltoblastus* | *delta* | e60014 (25) | Sonnebait | Toenioen Eno | Amanoebang | 60A34 |
| *Deltoblastus* | *delta* | e30930 | Sonnebait | Basleo | Amanoebang | 60A33 |
| *Deltoblastus* | *delta* | e59230 | Sonnebait | Neoetpantoekak | Amanoebang | 60A34 |
| *Deltoblastus* | *delta* | e30929 | Sonnebait | Basleo | Amanoebang | 60A33 |
| *Deltoblastus* | *delta* | e59723 | Sonnebait | Basleo | Amanoebang | 60A34 |
| *Deltoblastus* | *delta* | e30250 | ? | kuafeu | Amarimi | 60A36 |
| *Deltoblastus* | *delta* | e59689 | Sonnebait | Neoetpantoekak | Amanoebang | 60A34 |
| *Deltoblastus* | *delta* | e30976 | Sonnebait | Kiomoko | Amanoebang | 60A33 |
| *Deltoblastus* | *delta* | e59845 | Sonnebait | Kioemoko | Amanoebang | 60A34 |
| *Deltoblastus* | *delta* | e59202 | Sonnebait | Neoetpantoekak | Amanoebang | 60A34 |
| *Deltoblastus* | *delta* | e30910 | Sonnebait | Basleo | Amanoebang | 60A33 |
| *Deltoblastus* | *delta* | e60014 (25) | Sonnebait | Toenioen Eno | Amanoebang | 60A34 |
| *Deltoblastus* | *delta* | e59685 | Sonnebait | Neoetpantoekak | Amanoebang | 60A34 |
| *Deltoblastus* | *delta* | e59942 | Sonnebait | Toenioen Eno | Amanoebang | 60A35 |
| *Deltoblastus* | *delta* | e59722 | Sonnebait | Basleo | Amanoebang | 60A34 |
| *Deltoblastus* | *delta* | e30927 | Sonnebait | Basleo | Amanoebang | 60A33 |
| *Deltoblastus* | *delta* | e59233 | Sonnebait | Neoetpantoekak | Amanoebang | 60A34 |
| *Deltoblastus* | *delta* | e59855 | Sonnebait | Kioemoko | Amanoebang | 60A34 |
| *Deltoblastus* | *delta* | e30921 | Sonnebait | Basleo | Amanoebang | 60A33 |
| *Deltoblastus* | *delta* | e59791 | Sonnebait | Soem Peh | Amanoebang | 60A34 |
| *Deltoblastus* | *delta* | e59208 | Sonnebait | Neoetpantoekak | Amanoebang | 60A34 |
| *Deltoblastus* | *delta* | e59228 | Sonnebait | Neoetpantoekak | Amanoebang | 60A34 |
| *Deltoblastus* | *delta* | e59947 | Sonnebait | Toenioen Eno | Amanoebang | 60A35 |
| *Deltoblastus* | *delta* | e59843 | Sonnebait | Kioemoko | Amanoebang | 60A34 |
| *Deltoblastus* | *delta* | e59720 | Sonnebait | Basleo | Amanoebang | 60A34 |
| *Deltoblastus* | *delta* | e30713 | Sonnebait | Faoet Ao | Amanoebang | 60A33 |
| *Deltoblastus* | *delta* | e30971 | Sonnebait | Kiomoko | Amanoebang | 60A33 |
| *Deltoblastus* | *delta* | e60033 | Sonnebait | ? | ? | 60A34 |
| *Deltoblastus* | *delta* | e59196 | Sonnebait | Neoetpantoekak | Amanoebang | 60A33 |
| *Deltoblastus* | *delta* | e59943 | Sonnebait | Toenioen Eno | Amanoebang | 60A35 |
| *Deltoblastus* | *delta* | e30935 | Sonnebait | Basleo | Amanoebang | 60A33 |
| *Deltoblastus* | *delta* | e30926 | Sonnebait | Basleo | Amanoebang | 60A33 |
| *Deltoblastus* | *delta* | e59789 | Sonnebait | Soem Peh | Amanoebang | 60A34 |
| *Deltoblastus* | *delta* | e30975 | Sonnebait | Basleo | Amanoebang | 60A33 |
| *Deltoblastus* | *delta* | e30974 | Sonnebait | Kiomoko | Amanoebang | 60A33 |
| *Deltoblastus* | *delta* | e30984 | Sonnebait | Kiomoko | Amanoebang | 60A33 |
| *Deltoblastus* | *delta* | e59728 | Sonnebait | Basleo | Amanoebang | 60A34 |
| *Deltoblastus* | *delta* | e59411 | ? | Netoe Kot | ? | 60A34 |
| *Deltoblastus* | *delta* | e59721 | Sonnebait | Basleo | Amanoebang | 60A34 |
| *Deltoblastus* | *delta* | e59941 | Sonnebait | Toenioen Eno | Amanoebang | 60A35 |
| *Deltoblastus* | *delta* | e30973 | Sonnebait | Kiomoko | Amanoebang | 60A33 |
| *Deltoblastus* | *delta* | e30949 | Sonnebait | Kioemoko | Amanoebang | 60A34 |
| *Deltoblastus* | *delta* | e30924 | Sonnebait | Basleo | Amanoebang | 60A33 |
| *Deltoblastus* | *delta* | e59946 | Sonnebait | Toenioen Eno | Amanoebang | 60A35 |
| *Deltoblastus* | *delta* | e59793 | Sonnebait | Soem Peh | Amanoebang | 60A34 |
| *Deltoblastus* | *delta* | e59000 | Sonnebait | Toenioen Eno | Amanoebang | 60A35 |
| *Deltoblastus* | *delta* | e59199 | Sonnebait | Neoetpantoekak | Amanoebang | 60A34 |
| *Deltoblastus* | *delta* | e30905 | Sonnebait | Basleo | Amanoebang | 60A33 |
| *Deltoblastus* | *delta* | e59682 | Sonnebait | Neoetpantoekak | Amanoebang | 60A34 |
| *Deltoblastus* | *delta* | e30714 | Sonnebait | Faoet Ao | Amanoebang | 60A33 |
| *Deltoblastus* | *delta* | e59210 | Sonnebait | Neoetpantoekak | Amanoebang | 60A34 |
| *Deltoblastus* | *delta* | e59893 | Sonnebait | Faoet Ao | Amanoebang | 60A33 |
| *Deltoblastus* | *delta* | e30970 | Sonnebait | Kiomoko | Amanoebang | 60A33 |
| *Deltoblastus* | *delta* | e30476 | Sonnebait | Kioemoko | Amanoebang | 60A34 |
| *Deltoblastus* | *delta* | e59844 | Sonnebait | Kioemoko | Amanoebang | 60A34 |
| *Deltoblastus* | *delta* | e59001 | Sonnebait | Toenioen Eno | Amanoebang | 60A35 |
| *Deltoblastus* | *delta* | e30972 | Sonnebait | Kiomoko | Amanoebang | 60A33 |
| *Deltoblastus* | *delta* | e59003 | Sonnebait | Toenioen Eno | Amanoebang | 60A35 |
| *Deltoblastus* | *delta* | e30982 | Sonnebait | Kiomoko | Amanoebang | 60A33 |
| *Deltoblastus* | *delta* | e59194 | Sonnebait | Neoetpantoekak | Amanoebang | 60A33 |
| *Deltoblastus* | *delta* | e59004 | Sonnebait | Toenioen Eno | Amanoebang | 60A35 |
| *Deltoblastus* | *delta* | e30981 | Sonnebait | Kiomoko | Amanoebang | 60A33 |
| *Deltoblastus* | *delta* | e30914 | Sonnebait | Basleo | Amanoebang | 60A33 |
| *Deltoblastus* | *delta* | e59193 | Sonnebait | Neoetpantoekak | Amanoebang | 60A33 |
| *Deltoblastus* | *delta* | e59002 | Sonnebait | Toenioen Eno | Amanoebang | 60A35 |
| *Deltoblastus* | *delta* | e59005 | Sonnebait | Toenioen Eno | Amanoebang | 60A35 |
